# A genome-wide scan for quantitative trait loci affecting limb bone lengths and areal bone mineral density of the distal femur in a White Duroc × Erhualian F_2 _population

**DOI:** 10.1186/1471-2156-9-63

**Published:** 2008-10-08

**Authors:** Huirong Mao, Yuanmei Guo, Guangcheng Yang, Bin Yang, Jun Ren, Sanfeng Liu, Huashui Ai, Junwu Ma, Bertram Brenig, Lusheng Huang

**Affiliations:** 1 Key Laboratory for Animal Biotechnology of Jiangxi Province and the Ministry of Agriculture of China, Jiangxi Agricultural University, Nanchang 330045, PR China; 2 Institute of Veterinary Medicine, Georg-August-University of Göttingen, Burckhardtweg 2, 37077 Göttingen, Germany

## Abstract

**Background:**

Limb bone lengths and bone mineral density (BMD) have been used to assess the bone growth and the risk of bone fractures in pigs, respectively. It has been suggested that limb bone lengths and BMD are under genetic control. However, the knowledge about the genetic basis of the limb bone lengths and mineralisatinon is limited in pigs. The aim of this study was to identify quantitative trait loci (QTL) affecting limb bone lengths and BMD of the distal femur in a White Duroc × Erhualian resource population.

**Results:**

Limb bone lengths and femoral bone mineral density (fBMD) were measured in a total of 1021 and 116 F_2 _animals, respectively. There were strong positive correlations among the lengths of limb bones and medium positive correlations between the lengths of limb bones and fBMD. A whole-genome scan involving 183 microsatellite markers across the pig genome revealed 35 QTL for the limb bone lengths and 2 for femoral BMD. The most significant QTL for the lengths of five limb bones were mapped on two chromosomes affecting all 5 limb bones traits. One was detected around 57 cM on pig chromosome (SSC) 7 with the largest *F*-value of more than 26 and 95% confidence intervals of less than 5 cM, providing a crucial start point to identify the causal genes for these traits. The Erhualian alleles were associated with longer limb bones. The other was located on SSCX with a peak at 50–53 cM, whereas alleles from the White Duroc breed increased the bone length. Many QTL identified are homologous to the human genomic regions containing QTL for bone-related traits and a list of interesting candidate genes.

**Conclusion:**

This study detected the QTL for the lengths of scapula, ulna, humerus and tibia and fBMD in the pig for the first time. Moreover, several new QTL for the pig femoral length were found. As correlated traits, QTL for the lengths of five limb bones were mainly located in the same genomic regions. The most promising QTL for the lengths of five limb bones on SSC7 merits further investigation.

## Background

Bone length and bone mineral density (BMD) are generally regarded as two important parameters to assess the bone growth in pigs [1]. Individuals with longer limb bones usually have taller body heights or heights at shoulder. It has been shown that the body height or height at shoulder is negatively correlated with backfat thickness, and the height at shoulder is an important item in determining the yield of ham, loin, picnic shoulder and shoulder butt [2,3]. Visual selection for moderate length of the leg combined with appropriate body length can improve structural soundness and decrease the economic loss resulted from structural unsoundness for producers in pigs [4].

Longitudinal growth of the skeleton occurs through the action of chondrocytes in the proliferative and hypertrophic zones of the growth plate [5]. The cellular and biochemical processes influencing endochondral bone growth are complex and not yet fully elucidated. So far, there are few genetic studies on limb bone lengths in pigs. To our knowledge, only four quantitative trait loci (QTL) affecting femur dimensions has been detected on pig chromosomes (SSC) 2, 4, 16 and 17 [6]. Studies in mice [7-10] and chickens [11] have shown that the limb bone lengths are strongly controlled by genes. Elucidation of the genetic basis of bone growth will provide insight to understanding of the pathways and molecules involved in endochondral growth [12]. Bone mineral density (BMD) is a major determinant of risk for osteoporosis and bone fracture, which commonly affect the bone qualities of the distal forearm, thoracic and lumbar vertebrae, and proximal femur [13]. BMD is a complex trait with a high heritability, ranging from 0.5 to 0.9 in humans [14] and 0.6 to 0.7 in mice [15,16]. Furthermore, 60–85% and 80% of the phenotypic variance of BMD are genetically determined in humans [17] and mice [15], respectively. More recently, genome-wide scans for QTL affecting BMD have been widely performed in humans [18-20] and mice [15,16,21], and a number of QTL for BMD at different bone sites were mapped. In contrast, few QTL studies have been performed in farm animals [22], and none of QTL study was reported in pigs.

Because the physiologies are similar between pigs and humans, the pig is an ideal animal model for studying the genetic basis of bone growth and osteoporosis in humans, such as osteonecrosis of the femoral head, fractures of cartilage and bone, bone ingrowth [23,24]. The purpose of this study was to identify genomic regions affecting the lengths of limb bones (scapula, ulna, tibia, humerus and femur) and BMD of the distal femur in a large F_2 _resource population.

## Results

### Descriptive statistics of traits and correlations between traits

The descriptive statistics of the traits measured are listed in Table 1. Each limb bone was longer on average in castrated males than intact females. The correlation coefficients between the traits are shown in Table 2. All of the correlations were positive and highly significant (*P *< 0.001), especially for those among the lengths of limb bones. The BMD of the distal femur (fBMD) showed medium correlations with the lengths of limb bones.

**Table 1:** Descriptive statistics of limb bones lengths and BMD of the distal femur in the White Duroc × Erhualian intercross

Trait^a^	No.	Mean	Standard deviation	Min	Max
All					
Scapula length (SL), cm	1021	22.39	1.55	17.7	26.9
Ulna length (UL), cm	1020	18.33	1.25	14.6	22.3
Humerus length (HL), cm	1021	20.06	1.50	14.1	24.8
Femur length (FL), cm	1020	20.94	1.31	14.8	24.7
Tibia length (TL), cm	1017	18.83	1.22	15.3	22.7
Castrated males					
Scapula length (SL), cm	543	22.58	1.51	17.7	26.9
Ulna length (UL), cm	543	18.68	1.24	15.3	22.3
Humerus length (HL), cm	543	20.43	1.46	16.0	24.8
Femur length (FL), cm	543	21.12	1.29	14.8	24.7
Tibia length (TL), cm	542	18.97	1.21	15.3	22.7
BMD of the distal femur (fBMD), g/cm^2^	116	1.314	0.149	0.699	1.537
Intact females					
Scapula length (SL), cm	478	22.17	1.57	17.7	26.9
Ulna length (UL), cm	477	17.94	1.15	14.6	20.9
Humerus length (HL), cm	478	19.64	1.43	14.1	24.5
Femur length (FL), cm	477	20.72	1.31	17.3	24.0
Tibia length (TL), cm	475	18.66	1.21	15.4	21.9

^a. ^Abbreviations are given in the parentheses.

**Table 2:** Phenotypic correlation coefficients among limb bone lengths and fBMD^a^

	FL	TL	HL	UL	fBMD
SL	0.8310	0.8572	0.8831	0.8361	0.3141
FL		0.8984	0.8849	0.8164	0.3122
TL			0.9061	0.8709	0.3210
HL				0.8893	0.3318
UL					0.3232

^a ^For abbreviations see Table 1; The correlation coefficients are listed in the upper triangle, and all *P *values of the correlation coefficients are lower than 0.0001 except for fBMD (*P *< 0.001).

### QTL for limb bone lengths

**Figure 1:**
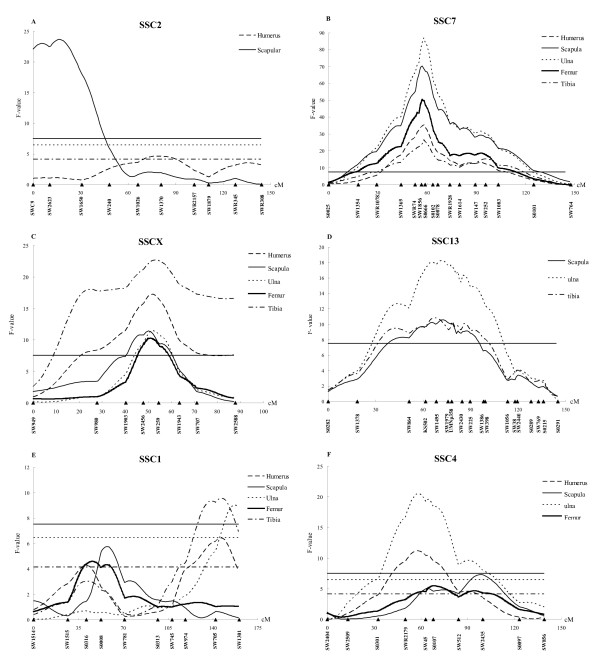
**Evidence of significant QTL for lengths of the limb bone on pig chromosomes 2 (A), 7 (B), X (C), 13 (D), 1 (E) and 4 (F). **The relative positions in cM on the linkage map are indicated in the x-axis, and the *F *values are given in the y-axis. Three lines are provided for 1% genome-wide (―), 5% genome-wide (----), and suggestive significance (– - – - – -) levels.

The results of QTL analysis are given in Table 3. The threshold values of 1% and 5% genome-wise significant and suggestive QTL for limb bone lengths were 7.53, 6.49, and 4.17, respectively. A total of 35 QTL were detected for the lengths of limb bones, and the number of QTL for each trait ranged from five (TL) to nine (SL). Due to the strong correlations among the limb bone lengths (Table 2), most of QTL regions showed associations with the lengths of two or more limb bones. Moreover, most of QTL had highly significant additive effects, but five QTL had significant dominance effects and a paternally expressed QTL affecting SL at 1% genome-wide significant level was found at 17 cM on SSC2 (Fig. 1A and Table 3).

Two QTL at 1% genome-wide significant level were found for the lengths of five limb bones (Table 3). One was located around 57 cM flanking by *SW1856 *and *S0102 *on SSC7, explaining 7 to 20% of the phenotypic variance (Fig. 1B). This QTL was the most significant QTL detected with small 95% confidence intervals (CI_95_) ranging from 2.5 (FL) to 5 cM (TL). The Erhualian alleles increased bone lengths at this locus. The other was in a region flanked by *SW2456 *and *SW1943 *on SSCX, explaining 2 to 6% of the phenotypic variance (Fig. 1C). At this locus, alleles from the White Duroc breed were associated with longer limb bones, and the CI_95 _varied from 12 (FL) to 59.5 cM (TL).

Three 1% genome-wide significant QTL each for SL, UL and TL were detected in an interval from 67 to 72 cM on SSC13 (Figure. 1D and Table 3). The favorable alleles were inherited from the White Duroc breed. A 1% genome-wide significant QTL for TL was found at the distal end of SSC1. In the same region, a 1% genome-wide significant QTL for UL and a 10% genome-wide significant QTL for HL were detected. Favorable QTL alleles at these loci were from the Erhualian breed. A suggestive QTL for SL and FL was evidenced at a different position on this chromosome (Fig. 1E and Table 3).

**Table 3:** The QTL mapping results for the lengths of limb bones and fBMD

SSC^a^	Position (cM)	Trait^b^	*F *Value^c^	Origin^d^	ADD ± S.E.^e^	DOM ± S.E.^e^	IMP ± S.E.^e^	CI_95_^f^	Var%^g^	Nominal *P*
1	46	FL	4.6*	Erhualian	-0.08 ± 0.03	-0.10 ± 0.04	ns	34–152	1.1	3.3E-03
	58	SL	5.8*	Duroc	ns	-0.24 ± 0.06	ns	0–113	1.5	6.7E-04
	146	HL	6.4*	Erhualian	-0.12 ± 0.03	ns	ns	27–153	1.9	2.6E-04
	147	TL	9.6***	Erhualian	-0.16 ± 0.03	ns	ns	36–160	1.0	3.2E-06
	157	UL	9.0***	Erhualian	-0.14 ± 0.03	-0.11 ± 0.05	ns	146–160	2.4	6.8E-06
2	17	SL	23.7***	Duroc	0.13 ± 0.04	ns	0.27 ± 0.04	0–23	5.4	9.7E-15
	78	HL	4.7*	Duroc	ns	ns	-0.11 ± 0.03	0–144	1.0	3.0E-03
3	40	FL	4.9*	Duroc	ns	-0.24 ± 0.07	ns	31–128	1.5	2.4E-03
	114	SL	7.3**	Erhualian	-0.17 ± 0.04	ns	ns	31–123	1.0	8.3E-05
4	58	HL	11.2***	Duroc	0.18 ± 0.03	ns	ns	46–85	1.5	3.2E-07
	58	UL	20.5***	Duroc	0.26 ± 0.03	ns	ns	54–75	2.8	7.3E-13
	68	FL	5.5*	Duroc	0.10 ± 0.03	ns	ns	3–109	4.5	9.2E-04
	98	SL	7.3**	Duroc	0.16 ± 0.04	ns	ns	0–110	1.3	7.8E-05
	127	fBMD	4.9*	Erhualian	-0.04 ± 0.02	-0.07 ± 0.03	ns	0–139	10.3	3.4E-03
5	54	SL	5.4*	Erhualian	-0.13 ± 0.04	ns	ns	1–105	1.0	1.1E-03
	64	UL	5.6*	Erhualian	-0.11 ± 0.03	ns	ns	8–112	1.1	7.8E-04
7	57	FL	50.3***	Erhualian	-0.41 ± 0.03	ns	ns	56–59	16.5	5.6E-30
	57	SL	70.4***	Erhualian	-0.59 ± 0.04	ns	-0.06 ± 0.03	56–60	9.5	9.3E-41
	58	HL	35.6***	Erhualian	-0.34 ± 0.03	ns	ns	55–59	19.9	1.1E-21
	58	TL	26.5***	Erhualian	-0.29 ± 0.03	ns	ns	54–59	13.5	2.1E-16
	58	UL	87.1***	Erhualian	-0.55 ± 0.03	0.09 ± 0.04	ns	57–60	7.0	2.9E-49
10	41	FL	5.9*	Duroc	0.12 ± 0.03	ns	ns	0–108	1.3	5.8E-04
11	106	fBMD	6.5*	Erhualian	-0.06 ± 0.02	ns	ns	36–106	14.7	4.9E-04
13	67	TL	10.9***	Duroc	0.16 ± 0.03	ns	ns	31–98	2.3	5.3E-07
	72	SL	10.6***	Duroc	0.19 ± 0.03	ns	ns	40–93	4.0	7.5E-07
	72	UL	18.3***	Duroc	0.22 ± 0.03	ns	-0.06 ± 0.03	60–96	2.7	1.7E-11
14	36	UL	6.1*	Duroc	0.11 ± 0.03	ns	0.07 ± 0.03	28–102	1.8	4.2E-04
	51	HL	4.6*	Duroc	0.12 ± 0.03	ns	ns	18–102	1.0	3.3E-03
	88	SL	8.4***	Duroc	0.16 ± 0.04	ns	-0.12 ± 0.04	52–97	1.2	1.7E-05
15	44	TL	6.4*	Erhualian	-0.14 ± 0.03	ns	ns	24–117	2.0	2.8E-04
	63	HL	8.1**	Erhualian	-0.14 ± 0.03	ns	ns	30–74	1.5	2.4E-05
	56	FL	4.7*	Erhualian	-0.10 ± 0.03	ns	ns	7–114	1.0	3.1E-03
X	50	SL	11.4***	Duroc	0.17 ± 0.04	ns	-0.14 ± 0.04	35–57	2.5	2.4E-07
	51	FL	10.3***	Duroc	0.13 ± 0.03	ns	-0.09± 0.03	48–60	4.5	1.1E-06
	52	HL	17.3***	Duroc	0.23 ± 0.03	ns	ns	43–59	2.4	6.5E-11
	52	UL	11.5***	Duroc	0.26 ± 0.05	ns	ns	45–60	2.6	2.2E-07
	53	TL	22.7***	Duroc	0.26 ± 0.03	ns	0.08 ± 0.03	24–84	5.9	3.6E-14

^a ^Pig chromosome; ^b ^For abbreviations see Table 1; ^c ^Significance levels: * suggestive significant level, **5% genome-wide significant level, ***1% genome-wide significant level.; ^d ^Origin of favorable allele respect to the founder breed; ^e ^ns, insignificant ADD, DOM and IMP. ^f ^The 95% confidence interval;^g ^Percentage of phenotype variance explained by the QTL.

On SSC4 at 58 cM between *SWR2179 *and *SW45*, QTL was detected for HL and UL at 1% genome-wide significant level. At another region between *SW45 *and *S0097 *on this chromosome, two QTL were mapped for SL at 5% genome-wide significant level and for FL at suggestive level, respectively (Fig. 1F and Table 3). The White Duroc alleles were associated with longer HL, UL, SL and FL at these loci.

### QTL for areal BMD

The critical values for 1%, 5% genome-wide and suggestive significance levels of fBMD QTL were 11.3, 8.16 and 4.56, respectively. Only two suggestive QTL were detected for this trait, one was located at 106 cM on SSC11 and the other was at 127 cM on SSC4 (Table 3). The two QTL jointly explained 25% of phenotypic variance, having significant additive and dominance effects whereas no imprinting effect. The Erhualian alleles were the favorable alleles for fBMD at the two loci.

### Interaction between QTL and sex

Three of 37 QTL had significant interaction effects with sex (*P *< 0.05). One was a suggestive QTL for SL at 54 cM on SSC5 and the other two were 1% genome-wide significant QTL for UL at 58 cM and FL at 57 cM on SSC7, respectively (Table 4).

**Table 4:** The interactions between QTL and sex for limb bones

SSC^a^	Trait^b^	Position	*F *Value^c^	Sex	ADD ± S.E.^d^	DOM ± S.E.	IMP ± S.E.
5	SL	54	4.5*	Male	ns^e^	ns	ns
				Female	**-0.23 ± 0.05**	ns	ns
7	UL	58	45.8***	Male	**-0.57 ± 0.04**	ns	ns
				Female	**-0.53 ± 0.05**	*0.23 ± 0.06*	ns
7	FL	57	27.0***	Male	**-0.38 ± 0.04**	ns	ns
				Female	**-0.46 ± 0.05**	*0.14 ± 0.06*	ns

^a ^Pig chromosome;

^b. ^For abbreviations see Table 1;

^c ^Significance levels: *suggestive significant level, ***1% genome-wide significant level

^d ^Significant dominant and additive effects are indicated in italic (*P *< 0.05) and bold (*P *< 0.01), respectively.

^e ^ns, insignificant values.

## Discussion

In this study, significant segregation was observed in the bone-related traits measured in White Duroc × Erhualian intercross. Strong positive correlations among the lengths of five limb bones were found, indicating that causal genes have effects on general mechanisms of bone length growth. Accordingly, several QTL for the lengths of different limb bones were detected at the same positions, such as QTL on SSC7, SSCX and SSC13. A similar situation has been observed in the previous QTL analysis of limb bone lengths in mice [10].

A total of 37 QTL were evidenced including 35 QTL for the limb bone lengths and 2 QTL for fBMD. To our knowledge, it is the first time to report QTL for SL, HL, UL, TL, and fBMD in pigs. All QTL regions for FL, HL, TL and UL on autosomes are orthologous to the intervals harboring the loci for these long bones lengths in mouse models [7,9,10], whereas QTL for SL except that on SSC4 do not correspond to the SL QTL regions in mice [7]. QTL for femoral dimension have been detected on SSC2, 4, 16 and 17 in a Wild boar × Large White intercross [6], which was not repeated in this study. We instead found novel QTL for FL on SSC1, 3, 7, 10, 15 and X and a suggestive QTL for FL on SSC4 at a different position (35 cM vs 68 cM). The discordant QTL for FL are possibly caused by the different genetic background of founder animals in the two experimental populations or due to false discovery of QTL.

The QTL on SSC7 was the most significant QTL detected with a CI_95 _of less than 5 cM, providing a promising region to identify genes responsible for the length of limb bones. This genomic region is homologous to human chromosome (HSA) 6p21, which has significant effect on human stature by interaction with HSA2q21. A cluster of candidate genes for longitudinal or skeletal growth have been considered on HSA6p21, such as *RUNX2*/CBFA1 (*runt-related transcription factor 2*), *COL11A2 *(*collagen, type XI, alpha 2*) and *RXRB *(*retinoid × receptor, beta*) [25]. Another promising candidate gene is *SCUBE3 *(*signal peptide-CUB-EGF-like domain-containing protein 3*) that is proximal to the QTL peak and plays a critical role in bone cells with an exclusive expression in bones and osteoblasts [26]. Significant QTL for growth, carcass and fatness traits have been consistently mapped at positons close to the QTL region on SSC7 . It has been shown that body height has negative correlation with backfat thickness and has effect on the yield of ham, loin, picnic shoulder and shoulder butt [2,3]. The overlapping QTL region for limb bone length, growth and fatness traits indicates that there might be gene(s) that has pleiotropic effects on these traits in the region.

The QTL on SSCX is another region showing significant association with limb bone lengths. The region is homologous to HSAXq2.4, where a QTL for height and several QTL for syndromes of idiopathic short stature have been evidenced [27]. At the proximal end of the p-arm of SSC2, a 1% genome-wide significant QTL specific for SL was found with a significant maternal imprinting effect. *Insulin-like growth factor 2 *(*IGF2*) playing a major role in muscle growth and fat deposition is an interesting candidate gene for the QTL because its effect coincides with *IGF2 *imprinting effect. A single nucleotide substitution in *IGF2 *intron 3 has been identified to be the causative mutation for a major maternal-imprinting QTL in this region [28].

We only detected two suggestive QTLs for fBMD on SSC11 and SSC4, respectively. The QTL region on SSC4 is homologous to HSA1q21-23 encompassing a QTL for human spine BMD [19]. The QTL region on SSC11 is homologous to HSA13q32-34 harbouring a QTL for spine BMD [18] and a QTL for distal forearm areal BMD [20].

In this study, we performed QTL mapping by using combined-sex analyses. Taking the caution that QTL for these bone-related traits could be affected by sex, we analyzed the interaction effects between QTL and sex. Three QTL had significant interaction between QTL and sex. One was a suggestive QTL for SL on SSC5 that was expressed exclusively in intact females. The others were two genome-wide significant QTL each for UL and FL on SSC7, at which the dominant effect was expressed only in intact females. Nevertheless, these interaction effects only dropped the *F*-values without altering the evidence of QTL on this chromosome, confirming that the QTL mentioned above are reliable by the combined-sex analyses.

## Conclusion

This study detected a total of 37 QTL for the lengths of scapula, ulna, humerus and tibia and BMD of the distal femur in pigs for the first time. Moreover, several new QTL for the femoral length in pigs were found. As correlated traits, many QTL for the lengths of five limb bones were located in the same genomic regions. The most promising QTL for each limb bone length were all located on SSC7 with the largest *F*-values and the smallest confidence intervals. It merits fine mapping of the QTL or the identification of positional candidate genes. Alleles from the White Duroc breed were not systematically favorable for longer length of limb bones.

## Methods

### Animals

A three-generation resource population was developed and managed as describled in our previous report [29]. Briefly, two White Duroc boars and 17 Erhualian sows were mated to produce 9 F_1 _boars and 59 F_1 _sows. These F1 animals were then intercrossed avoiding sister-brother mating to generate a total of 1912 F2 animals in 6 batches. In this study, 116 castrated males were recorded for fBMD and 1021 F_2 _animals including castrated males and intact female were measured for limb bone lengths. All F_2 _piglets were weaned at 45 d of age, and the males were castrated at the age of three months. All animals were housed in half-open pens and fed with mineral enforced corn-soybean diets.

### Phenotype recording

Both forelimb and hind limb were removed from the right side carcass of F_2 _animals when slaughtered at 240 d of age. Five limb bones were dissected from the limbs and the lengths of these bones were measured with a large caliper: the scapula (the maximum straight line distance from the cavitas glenoidalis to the border of scapular cartilage), humerus (total length from the head to the trochlea), ulna (length from the olecranon process to the styloid process), femur (total length from the greater trochanter to the intercondyloid fossa) and the tibia (length from the intercondylar eminence to the medial malleolus). After that, the femur bones of the 116 F_2 _individuals in the first batch were stored at -20°C until utilized.

Areal bone mineral densities at the distal femur (fBMD) of the pigs were measured by a dual-energy X-ray absorptiometry (DXA) (Challenger, Montpellier, France) with a precision of ± 1.5%. At least 40% of each bone was scanned from the same end, and a common area was selected for fBMD measurement.

### Genotyping and genetic map construction

Genome DNA was extracted from ear chip or spleen tissue, and a total of 183 informative microsatellites distributing 19 porcine chromosomes were genotyped for 1828 pigs including 19 founder animals, 68 F_1 _animals and 1741 F_2 _pigs in the White Duroc × Erhualian intercross. The number of markers on each chromosome varies from five (SSC18) to 24 (SSC13). Linkage analyses were carried out using the Crimap2.4 package [30]. Recombination units were transformed to map distances using the Haldane mapping function, and then the linkage maps were constructed.

### Statistical analysis

Descriptive statistics of the traits measured in the White Duroc × Erhualian intercross and the correlation coefficients between traits were calculated by the MEANS and CORR procedures of SAS Version 9.0 (SAS Institute Inc., Cary, USA). The GLM procedure of SAS was employed to determine the fixed effects and the covariates in the final QTL mapping model. Factors having significant effect on traits were included in the QTL analysis model.

A least squares regression method was used to perform QTL analysis using QTLexpress at [31]. The underlying assumption of this method is that the alternative alleles at a given QTL are fixed in the founder breeds. The two QTL alleles were defined as D from White Duroc and E from Erhualian in this study. There are four possible genotypes (DD, DE, ED and EE) at each analysis point. The probabilities of the four QTL genotypes, viz. Prob(DD), Prob(DE), Prob(ED) and Prob(EE), for each F2 animal were inferred from the flanking markers at each centi-Morgan (cM) and the coefficients of additive, dominance and imprinting effects were equal to Prob(DD) minus Prob(EE), Prob(DE) plus Prob(ED) and Prob(DE) minus Prob(ED), respectively. Finally, the phenotypic values were regressed onto these coefficients to estimate the QTL effects at each cM across the genome. Family and sex were included in all QTL models as fixed effect. Batch and carcass length were considered respectively as fixed effect and covariate in the models for the lengths of limb bones. Additionally, some other special factors were also considered in the final model for QTL mapping as fixed effects or covariates including sex as fixed effect in analysis UL, live weight as a covariate in SL, FL and aBMD, and the age at slaughter as a covariate in SL. For analyses of the X chromosome, only the phenotypic data of female F2 progeny was considered in the QTL analysis like autosomal QTL scans.

To investigate whether the QTL had different effects between males and females, the interaction between QTL and sex was tested. Each detected QTL was fixed at the same position and reanalyzed with an interaction model. If the interaction model was significantly better than the non-interaction model, the interaction between QTL and sex was considered. The statistic of the test followed an F distribution, and the F value is the ratio of the deviation of the residual sum of squares (RSS) of the non-interaction model from the RSS of the interaction model to the residual mean square (MS) of the interaction model. The numerator and the denominator degree of freedom were equal to 3 (gender fixed in the non-interaction model) or 4 (gender not fixed in the non-interaction model) and the population size minus the number of effects was fixed in the interaction model, respectively.

A permutation method was used to get the empirical distribution of the test statistic by 1,000 times permutation [32], and the critical values were obtained based on the empirical distribution. The 95% confidence interval of QTL was constructed by a bootstrap method [33]. Based on 2,000 replacement resample, the empirical distribution of the QTL position was obtained and the 95% confidence interval was determined. Percentage of variance explained by each QTL was calculated using the following formula:

$$Var\%=\frac{\left(MS_{reduce1}-MS_{full}\right)}{MS_{reduce}}\times 100$$

Where *MS*_full_, *MS*_reduce1 _and *MS*_reduce _were the mean squares of the models with all detected QTL, with the rest detected QTL except for the current focused one, and without all of the detected QTL, respectively.

## Authors' contributions

HRM performed marker genotyping with BY and prepared the draft with YMG. YMG finished all of the data analysis, and recorded phenotypes with GCY and HSA. JR made a major revision of the manuscript. BB revised the draft. LSH conceived the research plan and made a final revision of the manuscript. All authors read and approved the final manuscript.
